# Maternal health literacy and its associated factors among pregnant women in Western Cape antenatal clinics

**DOI:** 10.4102/phcfm.v18i1.5436

**Published:** 2026-08-13

**Authors:** Thabani M. Noncungu, Talitha Crowley, Jennifer A. Chipps

**Affiliations:** 1 School of Nursing, Faculty of Community and Health Sciences, University of the Western Cape, Bellville, South Africa

**Keywords:** antenatal clinics, decision-making behaviour, maternal health literacy, pregnant, women

## Abstract

**Background:**

Maternal health literacy influences how pregnant women access, understand and use healthcare services for themselves and their children. Limited maternal health literacy is associated with poor maternal and foetal outcomes.

**Aim:**

To assess maternal health literacy and its associated factors among pregnant women attending antenatal clinics in the Western Cape.

**Setting:**

The study was conducted in antenatal clinics of the Western Cape.

**Methods:**

A cross-sectional survey was conducted among 248 pregnant women using the Maternal Health Literacy Inventory in Pregnancy (MHELIP), together with measures of socio-demographics, depression and functional health literacy (reading and understanding). Data were analysed using the Statistical Package for Social Sciences (SPSS) version 29. Descriptive statistics summarised participant characteristics and maternal health literacy domains, while non-parametric tests and logistic regression identified predictors of maternal health literacy.

**Results:**

Participants had a mean age of 27.4 years (± 7.53; range: 18-45), and 60.5% (*n* = 150) reported more than two pregnancies. Most respondents (80.0%, n = 196) had limited maternal health literacy, with a mean MHELIP score of 57.8 out of 100 (standard deviation [s.d.]: 10.8; range: 21–87). Adequate maternal health literacy was significantly associated with higher reading, understanding, and decision-making scores, while women with no risk of depression scored higher in assessing maternal health information (*p* < 0.05). Reading and understanding significantly predicted maternal health literacy (odds ratio [OR]: 1.20), explaining 10.0% of the variance.

**Conclusion:**

Most pregnant women had limited maternal health literacy, underscoring the need for tailored antenatal education.

**Contribution:**

The findings inform the development of maternal health literacy programmes to support informed decision-making among pregnant women in South Africa.

## Introduction

More than 700 women died from preventable causes related to pregnancy and childbirth every day in 2023.^1^ A substantial global reduction of around 40% in the maternal mortality ratio (MMR) was observed between 2000 and 2023.^1^ Yet far below the Sustainable Development Goals of less than 70 per 100 000 by 2030.^1,2,3^ In South Africa, the MMR was estimated at 118 deaths per 100 000 live births in 2023, representing an 8.5% decrease from 2022, when the MMR was reported at 129 deaths per 100 000 live births.^4^ Despite this improvement, maternal mortality remains unacceptably high. Some adverse pregnancy outcomes may be attributed to low health literacy,^5^ which limits women’s ability to make informed health decisions and, in turn, compromises healthcare outcomes for the individual and her family.^6^

### Maternal health literacy

Health literacy is the degree to which individuals have the capacity to obtain, understand, process and apply basic health information and services needed to make appropriate health decisions. Individuals with adequate levels of health literacy can take responsibility for their health and that of those in their care.^7,8,9^ Maternal health literacy is defined as the ability of pregnant women to access, understand, process and use health information to make decisions and promote self-care during pregnancy.^10^ Several systematic reviews have found that the ability to access, understand, process and use health information is associated with having adequate health literacy and is necessary for improving and maintaining good health during pregnancy.^11,12^ Maternal health literacy encompasses several key components, including knowledge of maternal health conditions, the ability to seek and access maternal health information, critically appraising or assessing such information and applying it effectively in informed health decision-making and behaviour.

*Maternal health knowledge* is defined as a mother’s comprehension of critical health-related information across the stages of pregnancy, childbirth and the postpartum period. Maternal health knowledge includes awareness of maternal health conditions, appropriate health practices, recognition of danger signs, newborn care, nutrition and hygiene.^13,14^ *Searching* for maternal health information reflects a woman’s capacity and motivation to actively seek pregnancy-related health information from a variety of accessible sources.^15^ This behaviour involves identifying, locating and retrieving relevant information through channels such as healthcare professionals, printed materials, digital media, social networks and community resources.^16^ Proficient information-seeking behaviour empowers women to make informed decisions and engage in practices that support positive maternal and neonatal health outcomes.^17^ The *assessment* of maternal health information measures a woman’s ability to critically evaluate the accuracy, relevance, credibility and applicability of pregnancy-related information obtained from various sources.^18^ This cognitive skill involves discerning between trustworthy and misleading or conflicting information, assessing the qualifications of the source and determining whether the information aligns with evidence-based healthcare practices.

*Maternal health decision-making behaviour* measures a woman’s capacity to utilise health information to make informed choices regarding her own health and that of her child.^19^ This includes decisions related to antenatal care attendance, birth preparedness, choice of place of delivery, nutrition, hygiene practices, recognition and response to danger signs and care-seeking during pregnancy and postpartum. Maternal health literacy plays a crucial role in enabling pregnant women to make informed health-related decisions and maintain good health during pregnancy.^12,20,21^ Low maternal health literacy has been linked to numerous adverse outcomes, including unhealthy behaviours, higher mortality, delayed initiation of antenatal care, missed antenatal appointments and not attending pre-conception counselling.^22,23,24^ It is also associated with increased hospitalisations, greater use of emergency services, poor overall health status, lower self-efficacy, depression and smoking.^9,25,26^ For example, studies conducted in Australia and Turkey found that women with low reading skills and understanding were more likely to present with conditions such as diabetes, depression and asthma and were less likely to exclusively breastfeed or engage in early prenatal care compared to women with adequate health literacy.^27,28^

### Factors influencing maternal health literacy

Maternal health literacy is influenced by multiple factors, including individual characteristics such as educational attainment and literacy skills, and external and contextual factors, including socio-demographic conditions (e.g. income and household composition).^22,26,29,30^ Individual factors include psychological distress or depression, level of education^31^ and reading and writing abilities.^32^ The ability to read and comprehend health information is fundamental to achieving adequate maternal health literacy, as it enables women to understand health messages and make informed decisions.^11,33^ Maternal health literacy shows a significant inverse association with depression among pregnant women, such that higher levels of health literacy correspond to lower depression scores, while lower health literacy is linked to elevated depressive symptoms.^34,35^

Maternal depression can affect the ability to process information, make informed decisions and engage effectively with health-related guidance during pregnancy and early parenting.^36^ Conversely, limited health literacy may increase vulnerability to depressive symptoms, potentially limiting access to coping resources and understanding of health information and may also contribute to reduced health literacy.^37^

Maternal health literacy among pregnant women tends to be positively associated with educational attainment, with women who have higher levels of formal education generally demonstrating greater capacity to obtain, understand and apply health information related to pregnancy.^38^ Studies have shown that pregnant women with higher educational qualifications, such as university degrees, consistently higher score on health literacy measures compared with those who are illiterate or have lower levels of education.^22,39,40^ Conversely, lower educational attainment has been identified as a predictor of limited health literacy in antenatal populations, underscoring the importance of education in facilitating effective health communication and decision-making during pregnancy.^41^

Pregnancy-related factors arise during pregnancy and can influence women’s health behaviours and their access to care.^42^ These include the timing and frequency of antenatal care attendance, pregnancy-related complications (e.g. anaemia, hypertension or gestational diabetes) and adherence to recommended clinical and nutritional practices.^43,44,45^ Collectively, these pregnancy-related factors affect maternal health literacy and women’s ability to make informed pregnancy-related decisions, influencing maternal and neonatal outcomes. Globally, maternal health literacy is associated with education, socioeconomic status, age, employment and access to health information.^12,33^ In sub-saharan Africa, it is further influenced by poverty, limited healthcare access and weak health communication.^25^ In South Africa, disparities in education, socioeconomic status, language and access to maternal health information continue to influence maternal health literacy. However, limited evidence exists on how maternal health literacy interacts with individual, psychological socio-demographic and pregnancy-related factors in low-income South African settings. Hence, the current study investigates the associated factors among pregnant women attending antenatal clinics in the Western Cape.

## Research methods and design

### Study design

A quantitative survey was conducted using a researcher-administered questionnaire to measure the maternal health literacy of pregnant women attending antenatal clinics in the Western Cape, South Africa. This study was conducted in three selected antenatal clinics located in the Northern Tygerberg sub-district, Western Cape. The Northern Tygerberg sub-district is home to approximately 800 000 people from diverse races and economic classes.^46^

### Study population and sampling strategy

The study population comprised approximately 900 patients attending antenatal clinics during the data collection period in the Northern Tygerberg sub-district of the Western Cape. The sample size was calculated using a 5% margin of error, a 95% confidence level (*Z* = 1.96) and an assumed population proportion of 50% (*p* = 0.5) because of the absence of prior estimates. This yielded a required sample size of 267 participants.^47^ The inclusion criteria for the study were: Pregnant women older than 18 years, irrespective of their gravidity and parity. A systematic sampling approach was employed to recruit participants in a structured and practical manner. Based on an estimated population of 900 pregnant women and a required sample size of 267, a sampling interval of three was calculated by dividing the population size by the target sample. To ensure representative participation, a proportional sampling strategy was used across the three facilities by applying the systematic interval to the flow of patients at each site. The final sample included 89 participants from clinic 1, 89 from clinic 2 and 89 from clinic 3. Accordingly, every third eligible woman attending the facility was invited to participate. When a selected participant declined or was unavailable, the next eligible individual was approached in accordance with the predefined sampling procedure.

### Instrument

The study questionnaire included: (1) socio-demographics, including individual factors that can influence health literacy (age, level of education; awareness of gestational age); socio-demographic (employment status, household composition); pregnancy-related (gestational age), (2) the Maternal Health Literacy Inventory in Pregnancy (MHELIP) scale, (3) additional individual factors affecting literacy, that is depression and (4) reading and understanding ability (functional health literacy). The validated MHELIP scale, used with permission from the authors, consists of 48 statements to measure four domains of maternal health literacy, namely maternal health knowledge (21 items), searching (6 items), assessing (6 items) and maternal health decision-making behaviour (15 items) (Appendix 1). The MHELIP scale items were rated using a 5-point Likert scale: ‘I don’t know at all’ (1), ‘I know a little’ (2), ‘I know something’ (3), ‘I know a lot’ (4) and ‘I know fully’ (5). Based on the instructions for the MHELIP, the original scores were converted to a score from 0 to 100 using the following formula (Equation 1):


$$\text{score}:\frac{Raw\;score-Minimum\;possible\;raw\;score}{Maximum\;possible\;raw\;score-Minimum\;possible\;raw\;score}\times 100$$ [Eqn 1]

The converted scores were then classified into four categories: Inadequate (0–50), problematic (50.1–66), sufficient (66.1–84) and excellent (84.1–100). The inadequate and problematic health literacy levels were further grouped as limited health literacy (0–66) and sufficient and excellent literacy levels as desired health literacy (66.1–100). The MHELIP scale demonstrated moderate to good internal consistency in the original validation study (*α* = 0.94)^48^ and acceptable internal consistency in the current study (*α* = 0.84). The four subscales also showed acceptable reliability: maternal health knowledge (*α* = 0.78), search for maternal health information (*α* = 0.66), assessment of maternal health information (*α* = 0.57) and maternal health decision-making and behaviour (*α* = 0.69). Risk of depression was measured using two statements from the two-question case finding scale, designed to identify the risk of depression among respondents.^49^ The two statements were scored using a 2-point dichotomous (Yes or No) scale that ranges between No (0) and Yes (1). A ‘Yes’ response to either statement was indicative of a potential risk for depression. Reading and understanding of health information were measured using five (*n* = 5) statements from the Functional Communicative and Critical Health Literacy scale.^50^

The five statements measuring functional health literacy were scored using a 4-point Likert scale that ranges between never (1), almost never (2), sometimes (3) and often (4). To facilitate accurate analysis of the functional health literacy domain, items were reverse coded prior to analysis. Specifically, original response scores were transformed as follows: 1 to 4, 2 to 3, 3 to 2 and 4 to 1. This reverse coding was applied because the functional health literacy items were negatively phrased, ensuring that higher scores consistently reflected higher levels of health literacy across all domains. A higher total score (maximum = 20) on these items indicates a higher ability to read and understand information. The Cronbach alpha coefficient of the functional health literacy subscale demonstrated good internal consistency in a previous study, *α* = 0.84 (Ishikawa et al. 2008) and acceptable internal consistency in the present study (*α* = 0.77).

### Data collection

We collected data in 2022 over a period of 4 months between August 2022 and November 2022, through a paper-based questionnaire at the three antenatal clinics located in the Western Cape (South Africa). The questionnaire was translated from English into isiXhosa by African language experts from the Arts and Humanities Faculty. The respondents were presented with the study information about the study by researcher assistants and provided individual informed consent. The questionnaires were distributed by the researcher and two trained research assistants. The completion of the questionnaire took approximately 25 min. The study data were not shared with any individuals who were not directly involved in the conduct of the study.

### Statistical analysis

Descriptive statistics were employed to summarise the characteristics of the study variables and analyse the data. The study data were captured and cleaned in the Statistical Package for Social Sciences (SPSS) version 29.0 for analysis. The study examined the proposed predictions using the Mann–Whitney *U* test to assess the hypotheses. In addition, logistic regression was conducted, with the desired level of maternal health literacy specified as a binary outcome to assess the hypothesis of factors such as level of education and reading and understanding ability predicting maternal health literacy (Hypotheses 1 and 3). In addition, depression was included as the bi-directional relationship suggests that maternal depression can both stem from and contribute to reduced health literacy (Hypothesis 2):^37^

**H**_**1**_: There is a significant association between maternal health literacy and the level of education among pregnant women.

**H**_**2**_: There is a significant association between maternal health literacy and risk of depression among pregnant women.

**H**_**3**_: There is a significant association between maternal health literacy and reading and understanding among pregnant women.

### Ethical considerations

Ethical clearance to conduct this study was obtained from the University of the Western Cape Biomedical Science Research Ethics Committee (No. BM21/02/05).

## Results

### Socio-demographics

The study achieved a response rate of 92.8%, with 248 of the 267 eligible pregnant women participating. Nineteen questionnaires (7.2%) were removed because of incomplete completion of the key scales. No differences in key socio-demographic and obstetric variables were observed, suggesting a low likelihood of selection bias. The mean age of 248 respondents was 27.4 years (standard deviation [s.d.] = 7.53), ranging from 18 years to 45 years. Nearly all the respondents (*n* = 222, 89.5%) indicated that they had attended secondary education and higher. However, nearly three-quarters (*n* = 171, 69%) of the respondents had difficulty with reading and understanding instructions in pamphlets in the antenatal clinics, with a score of 14.00 out of 20.00 (s.d. = 3.92, range: 5–20) on the functional health literacy scale. More than half of the respondents (*n* = 127, 51.2%) were employed. Less than half (*n* = 107, 43.1%) of the respondents were living with a partner, over one-third (*n* = 79, 31.9%) were living with family and a quarter (*n* = 62, 25.0%) were living alone.

Nearly two-thirds of the respondents (*n* = 150, 60.5%) had more than two pregnancies, but only 122 of respondents (49.2%) were aware of the gestational age of their current pregnancy. Among the 122 respondents who were aware of their gestational age, more than two-thirds (*n* = 100, 40.3%) were in the third trimester of pregnancy (Table 1). A high proportion (just under half of the respondents, *n* = 106, 42.7%) were identified as being at risk of depression, indicating ‘yes’ on either of the two depression questions.

**TABLE 1:** Socio-demographic characteristics (*N* = 248).

Characteristics	Number	%	Mean	s.d.
**Age**	-	-	27.4	7.53
**Age (years)**	-	-	-	-
18–35	204	82.3	-	-
36–45	44	17.7	-	-
**Educational attainment**	-	-	-	-
Primary education	26	10.5	-	-
Secondary education and higher	222	89.5	-	-
**Gravidity**	-	-	-	-
< 1 pregnancies	98	39.5	-	-
> 2 pregnancies	150	60.5	-	-
**Knowledge of gestational age**	122	49.2	-	-
Gestational age	-	-	-	-
1st trimester	0	0.0	-	-
2nd trimester	22	8.9	-	-
3rd trimester	100	40.3	-	-
**Employment status**	-	-	-	-
Employed	127	51.2	-	-
Unemployed	94	37.9	-	
Student	27	10.9	-	-
**Household composition**	-	-	-	-
Living alone	62	25.0	-	-
Living with family	79	31.9	-	-
Living with partner	107	43.1	-	-
**Risk of depression**	106	42.7	-	-
**Reading and understanding**	-	-	14.0/20	3.92

s.d., standard deviation.

### Maternal health literacy

Maternal health literacy was measured with the MHELIP scale with its four domains to measure maternal health literacy, namely *Searching for maternal health information, Assessing maternal health information, maternal health knowledge* and *maternal health decision-making and behaviour*. The overall mean score for maternal health literacy was 57.80 out of 100 (standard deviation [s.d.]: 10.8; range: 21–87), with more than three-quarters (*n* = 196, 80.0%) of the respondents being classified as having limited levels of maternal health literacy. Of the 196 classified as having limited maternal health literacy, 149 (76%) were classified as having a problematic level of maternal health literacy (Table 2).

**TABLE 2:** Maternal health literacy scale (*N* = 248).

Scale	Limited (*n* = 196, 79.0%)				Desirable (*n* = 52, 21.0%)			
	Inadequate (0–50)		Problematic (50.1–66)		Sufficient (66.1–84)		Excellent (84–100)	
	*n*	%	*n*	%	*n*	%	*n*	%
Maternal health literacy	47	18.9	149	60.1	48	19.4	4	1.6

Note: Mean = 57.62/100, s.d. = 11.00, range: 21–100. Limited: Inadequate – Mean = 41.3/100, s.d. = 6.10; Limited: Problematic – Mean = 57.8/100, s.d. = 4.27; Desirable: Sufficient – Mean = 70.7/100, s.d. = 4.72; Desirable: Excellent – Mean = 6.4/100, s.d. = 1.52.

s.d., standard deviation.

No significant associations were found with depression, level of education, age, gravidity and household composition, but a significant association was identified between maternal health literacy and respondents’ reading and understanding of health information. Respondents with adequate maternal health literacy scored significantly higher in reading and understanding health information than those with limited maternal health literacy (15.12 vs. 13.71; *U* = 6270, *p* = 0.010).

### Maternal health literacy domains

**FIGURE 1:**
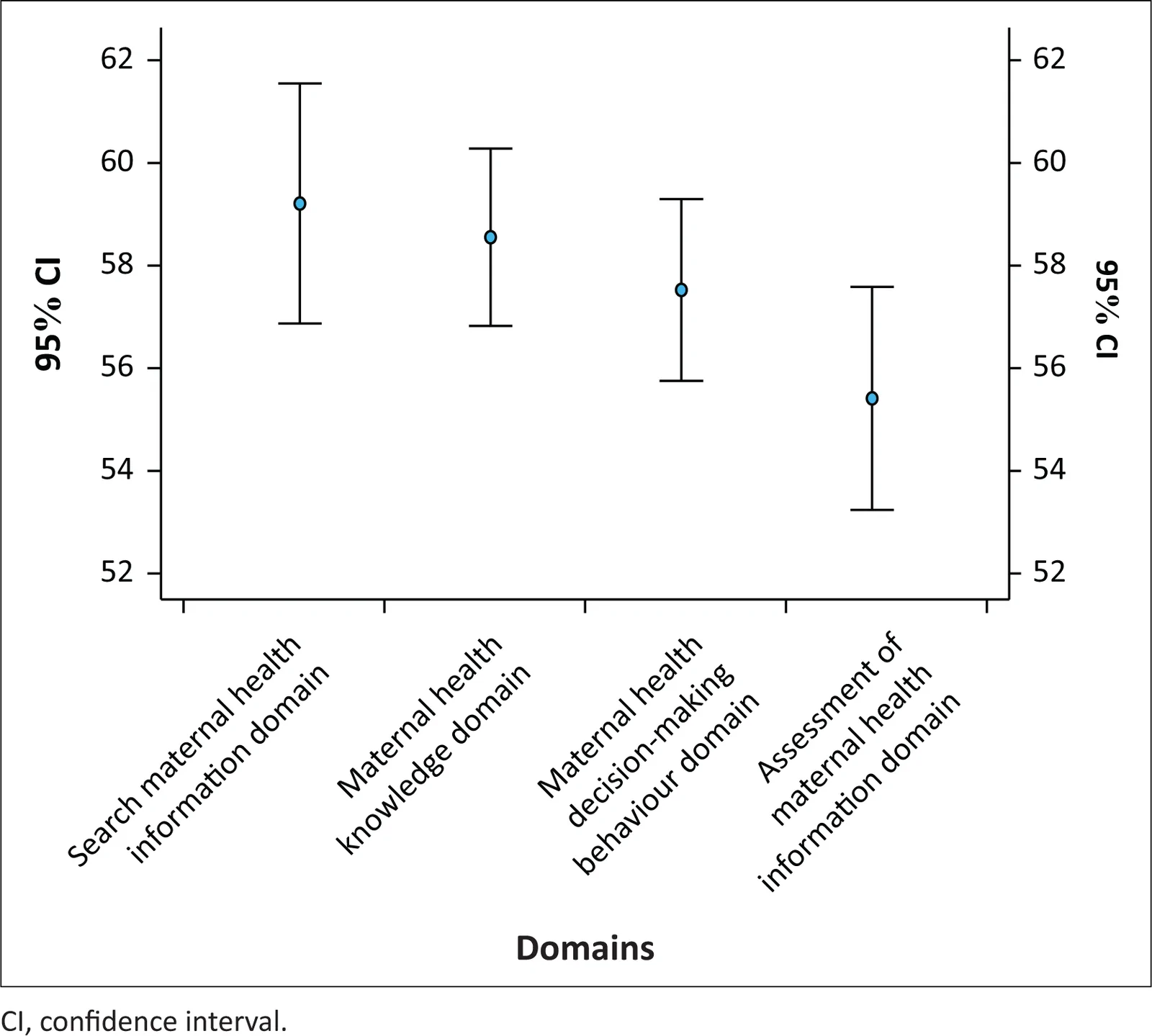
Maternal health literacy domains.

Across the maternal health literacy domains, the mean scores range from approximately 55 to 59 out of 100 (Figure 1). The *search maternal health information* domain was rated the highest (mean = 59.16/100, CI: 57–61), indicating comparatively stronger performance in information-seeking abilities, while assessment *of maternal health information* was rated the lowest (mean = 55.36/100, CI: 54–58). This indicates greater uncertainty and comparatively weaker skills in evaluating the quality and relevance of health information. These differences were, however, not significant (Figure 1).

### Searching for information related to maternal health

The average score for *searching for maternal health-related information* was 59.16 out of 100 (s.d.: 18.63; range: 13–100), with 150 (60%) respondents classified as having limited searching skills (Table 3). A Mann–Whitney *U* test revealed no statistically significant differences in the *Searching for Maternal Health Information* domain across respondents’ socio-demographic characteristics (level of education, gravidity and household composition), risk of depression or reported difficulty in reading and understanding health information.

**TABLE 3:** Distribution of maternal health literacy (*N* = 248).

Domains	Limited		Desirable	
	Inadequate	Problematic	Sufficient	Excellent
**Search maternal health information**				
*n*	88	62	80	18
%	35.5	25.0	32.3	7.3
Mean (59.16/100)	40.78/100	58.39/100	76.82/100	91.67/100
s.d. (18.63)	14.35	15.33	11.49	6.80
Range (13–100)	-	-	-	-
**Maternal health knowledge**				
*n*	55	127	60	6
%	22.2	51.2	24.2	2.4
Mean (58.50/100)	40.40/100	58.08/100	72.52/100	96.43/100
s.d. (13.79)	9.00	4.29	4.14	4.12
Range (13–100)	-	-	-	-
**Maternal health decision- making and behaviour**				
*n*	74	104	63	7
%	29.8	41.9	25.4	2.8
Mean (57.47/100)	40.50/100	59.12/100	66.91/100	82.50/100
s.d. (14.19)	12.50	10.23	11.14	13.15
Range (18–100)	-	-	-	-
**Assessment of maternal health information**				
*n*	108	70	57	13
%	43.5	28.2	23.0%	5.2
Mean (55.36/100)	40.96/100	53.89/100	70.83/100	93.75/100
s.d. (17.38)	14.54	13.62	14.51	17.38
Range (0–100)	-	-	-	-

s.d., standard deviation.

### Maternal health knowledge of pregnant women

The average score for maternal health knowledge was 58.50 out of 100 (s.d.: 13.79; range: 13–100), with nearly three-quarters of the respondents (*n* = 182, 73.4%) being classified as having inadequate to problematic (limited) levels of maternal health knowledge. Significant association was found between maternal health knowledge and level of education. Respondents with primary education demonstrated significantly higher maternal health knowledge scores compared with those with secondary education (65.43 vs. 57.69; *U* = 2147.00, *p* = 0.033).

### Maternal health decision-making and behaviour

The average score for *Maternal health decision-making and behaviour* was 57.47 out of 100 (s.d.: 14.19; range: 18–100), with nearly three-quarters of respondents (*n* = 178; 71.7%) classified as having inadequate to problematic (limited) levels of maternal health decision-making and behaviour. When reading and understanding health information (functional health literacy) was analysed across categories of decision-making skills in maternal health information, respondents with limited decision-making skills reported greater difficulty with reading and understanding health information compared to those with desirable decision-making skills. A statistically significant association was observed between maternal health decision-making behaviour and health literacy status, with respondents who had desired health literacy demonstrating significantly higher maternal health decision-making behaviour scores than those with limited health literacy (68.11 vs. 54.65; *U* = 7889, *p* < 0.001).

### Assessment of maternal health information

The average score for *Assessment of maternal health information* was 55.36 out of 100 (s.d.: 17.38; range: 0–100), which was the lowest-rated domain, with nearly three-quarters of the respondents (*n* = 178; 71.7%) classified as having inadequate to problematic (limited) levels of assessment of maternal health information in this domain. A significant association was observed between the ability to assess maternal health information and depression. Respondents with no risk of depression demonstrated significantly higher scores in assessing maternal health information compared with those at risk (57.54 vs. 52.44; *U* = 6296, *p* = 0.027).

### Factors that may impact maternal health literacy

Direct logistic regression was performed to assess the impact of the number of factors on maternal health literacy (limited vs. desirable). The model contained three independent variables (level of education, risk of depression and reading and understanding), which were significant in univariate testing. The full model containing all the predictors was significant, *χ*^2^ (3, *n* = 248) = 7.9, *p* = 0.048, indicating that the model was able to distinguish between the respondents with limited vs desirable maternal health literacy. The model explains between 3.1% (Cox and Snell *R*-squared) and 4.9% (Nagelkerke *R*-squared) of the variants in maternal health literacy and correctly classified 79.0% of the cases. As shown in Table 4, only one of the independent variables made a statistically significant contribution to the model (reading and understanding), recording an odds ratio of 1.2 (95% CI: 1.02–1.2), explaining 10.1% of the variance in maternal health literacy. The independent variables included in the logistic regression model of level of education, risk of depression and reading and understanding of health information were selected based on their statistical significance in the bivariate analyses. Maternal health literacy and depression are closely interconnected in a bi-directional relationship: Depression may impair a woman’s ability to access, understand and apply health information, while low health literacy may increase vulnerability to depressive symptoms.^35^ Variables that demonstrated a significant association with the dependent variable at the bivariate level (*p* < 0.05) were considered eligible for inclusion in the multivariable logistic regression model. This approach was adopted to ensure that only variables with meaningful unadjusted associations were retained while allowing the multivariable model to assess their independent effects after controlling for potential confounding.

**TABLE 4:** Logistic regression of maternal health literacy.

Variables	B	Sig.	Exp(B)	Lower	Upper
Level of education	−0.699	0.135	0.497	0.199	1.243
Risk of depression	0.219	0.492	1.245	0.666	2.33
Reading and understanding score	0.102	0.018	1.107	1.018	1.204
Constant	−2.277	0.002	0.103	-	-

CI, confidence interval; Sig. significant.

## Discussion

This study provides valuable insights into the level of maternal health literacy and the factors influencing it, specifically the level of education, risk of depression and functional health literacy (reading and understanding) among pregnant women attending three antenatal clinics in the Western Cape. Given the scarcity of studies of this nature within the South African context, these findings contribute important region-specific evidence to the existing body of literature. The respondents had low to moderate maternal health literacy (57.80/100; s.d.: 10.8; range: 21–87), which was lower than reported in studies conducted in other regions that utilised the same MHELIP scale.^48,51,52,53,54^ The observed differences in maternal health literacy scores may be attributed to variations in study settings and socio-demographic characteristics of the study populations. The current study found that more than three-quarters of respondents (80.0%) were classified as having limited maternal health literacy. This proportion is substantially higher than that reported in other settings; for example, a study conducted in Portugal found that only 47% of participants had limited health literacy, which was associated with factors such as lower educational attainment, being born outside the study country, and financial hardship.^55^ The highest mean score was observed for the domain ‘searching for maternal health–related information’ (59.16/100; s.d.: 18.63; range: 13–100) although 60.5% of participants were classified as having limited skills in this domain.

A significant association was observed between maternal health knowledge and level of education, with respondents possessing primary education demonstrating significantly higher maternal health knowledge scores compared with those with secondary education (65.43 vs. 57.69; *U* = 2147.00, *p* = 0.033). This unexpected finding suggests that formal education level does not always translate directly into maternal health knowledge. One possible explanation is that the small sample of primary-educated women (*n* = 26) compared to the larger secondary group (*n* = 222) suggests that the former may have been a more motivated, homogeneous group of information seekers, whereas the secondary group showed more varied levels of engagement. This finding is surprising, as we would normally expect respondents with secondary education to have higher maternal health knowledge scores than those with primary education, suggesting that formal education may not always translate into better maternal health knowledge. This may be attributed to the possibility that pregnant women with primary education actively seek maternal health information to compensate for their lower formal education, whereas those with secondary education may rely on prior knowledge or assume they are adequately informed. Furthermore, the markedly smaller proportion of respondents with primary education (*n* = 26/248), compared with the substantially larger group with secondary education (*n* = 222/248), may have influenced the findings. It is possible that all respondents with primary education actively sought maternal health information, whereas the larger secondary-educated group exhibited more heterogeneous health information–seeking behaviours.

To the best of our knowledge, this finding contrasts with the results of previous studies. For instance, a study conducted in South Africa reported that pregnant women with secondary education or higher scored higher on maternal health knowledge measures compared with those with only primary education.^56^ The low mean score observed for the assessment domain indicates that, although women may be able to access or read health information, many struggle to critically appraise this information and determine its applicability to their own maternal health needs. Participants experienced substantial challenges in evaluating the credibility, relevance and usefulness of maternal health information. Similar patterns have been reported in Hungary, where fewer than two-thirds of respondents demonstrated adequate capacity to assess maternal health information, reflecting a broader challenge in higher-order health literacy skills.^53^ In contrast, higher assessment scores reported in Turkey highlight potential contextual differences in education systems, health communication practices and support structures that may facilitate stronger evaluative skills.^57^

The fact that assessment was the lowest-rated maternal health literacy domain in the present study has important implications, as limited assessment skills may increase vulnerability to misinformation, hinder informed decision-making and reduce effective engagement with maternal health services. Furthermore, in the present study, respondents with limited assessment abilities reported greater difficulty in reading and understanding health information, suggesting that inadequate evaluative skills may exacerbate existing challenges in functional health literacy. These findings underscore the need for maternal health literacy interventions that extend beyond information provision and explicitly strengthen women’s capacity to critically assess maternal health information. The study found that the key predictor of maternal health literacy was functional health literacy, the ability to read and understand maternal health information.

The study found that for each one-unit increase in the reading and understanding score was associated with a 10.7% increase in the odds of having desirable maternal health literacy, indicating a modest but meaningful effect. Nearly three-quarters (69%) of the respondents reported having difficulty with reading and understanding instructions in pamphlets in the antenatal clinics. In contrast to the present study, evidence from Honduras indicates relatively high levels of functional health literacy, with more than 80% of participants reporting no difficulty understanding written health information and over half frequently comprehending disease-related information.^58^ This apparent contradiction highlights important contextual differences between settings rather than inconsistencies in measurement. In the South African context, lower levels of maternal health literacy may be influenced by structural and systemic factors, including linguistic diversity, mismatches between the language of health information and women’s home languages, variable quality of health communication within public healthcare facilities and educational inequalities. Additionally, written health materials may not be sufficiently tailored to local literacy levels or sociocultural contexts, limiting comprehension despite availability. These findings suggest that maternal health literacy in South Africa is shaped not only by individual reading ability but also by broader health system and communication factors, underscoring the need for contextually appropriate, plain language and multilingual maternal health education strategies.^59^

### Strength and limitations

This study provides baseline evidence on maternal health literacy among pregnant women in a South African setting where empirical research is limited and may inform future research, interventions and policy development to improve maternal health literacy. Respondents were recruited from three antenatal clinics in the Western Cape, which may limit the generalisability of the findings to pregnant women who do not attend antenatal care or who access private healthcare services. The achieved sample size was smaller than the minimum calculated sample size, which may have resulted in reduced statistical power and limited generalisability of the findings. Psychometric testing of the translated isiXhosa version of the questionnaire should be considered when interpreting the findings. Lastly, although this study provides some insights into the factors affecting maternal health in this population, other influential variables such as linguistic diversity, socioeconomic status and systemic healthcare access were not included in the final predictive model.

### Implications or recommendations

In response to low maternal health literacy scores, particularly in information assessment, searching and decision-making, together with the high risk of depression, antenatal services should integrate health literacy that has responsive education with routine mental health screening. Additionally, simplified language, visual aids and clear verbal communication are recommended to support women with reading and understanding difficulties.

## Conclusion

This study provided evidence of maternal health literacy and its associated factors in pregnant women. The current study found that pregnant women attending antenatal clinics in the Western Cape had a moderate level of maternal health literacy, which was lower than levels reported in other regions. Maternal health literacy was significantly associated with difficulty in reading and understanding health information, highlighting the importance of literacy-sensitive antenatal education to support informed maternal health decision-making. Furthermore, the findings suggest that formal education does not necessarily translate into superior maternal health knowledge. This highlights the need for targeted, context-specific health education interventions that go beyond formal schooling to strengthen maternal health literacy and support informed decision-making.

## Appendix 1

MHELIP scale: 48 Items.

### A. Maternal health knowledge

A1. I know natural physical changes during pregnancy.

A2. I know natural psychological changes during pregnancy.

A3. I know proper nutrition during pregnancy.

A4. I know personal health care.

A5. I know proper activity and status in pregnancy.

A6. I know proper exercise during pregnancy.

A7. I know pregnancy supplements (vitamins).

A8. I know the appropriate referral timing for pregnancy examinations (visits).

A9. I know diagnostic examinations (ultrasound and tests) of maternal and foetal health in pregnancy.

A10. I know the acceptable and normal amount of weight gain during pregnancy.

A11. I know common pregnancy problems such as nausea, vomiting and lower back pain.

A12. I know injecting safe (allowed) vaccines during pregnancy.

A13. I know the proper sexual relations during pregnancy.

A14. I know the normal number of foetal movements.

A15. I know the factors affecting foetal health such as photography, medications, chemicals such as botox, etc.

A16. I know risk signs in pregnancy.

A17. I know pregnancy disease symptoms such as gestational diabetes, high blood pressure in pregnancy and other diseases.

A18. I know childbirth such as the advantages and disadvantages of each of the natural delivery methods and caesarean section and their associated care

A19. I know the methods of pain relief in vaginal delivery.

A20. I know neonatal and infant care in the postpartum period.

A21. I know required postpartum care of mother.

### B. Searching maternal health information

B1. I acquire information from written materials such as books, educational notes, pamphlets and medication brochures.

B2. I acquire information from radio and television.

B3. I acquire information from Internet sources such as websites, Instagram and Telegram.

B4. I acquire information from other pregnant women.

B5. I acquire information from family, friends and acquaintances.

B6. I acquire information from healthcare professionals such as a physician or midwife.

### C. Assessment for maternal health information

C1. It is easy for me to read and pronounce pregnancy-related vocabulary from information sources such as books, educational booklets, Internet, Telegram and Instagram.

C2. The information obtained from different sources is understandable for me.

C3. I know valid and verified sources for getting the right pregnancy-related information.

C4. I ask of the doctor or midwife to make sure pregnancy-related information.

C5. I evaluate the accuracy of pregnancy-related information obtained from online sources such as websites, Instagram and Telegram.

C6. I evaluate the accuracy of pregnancy-related information obtained from friends and relatives.

### D. Maternal health decision-making and behaviour

D1. I am able to control/manage physical and psychological changes in pregnancy.

D2. I implement a proper diet for pregnancy.

D3. I implement necessary measures for personal health care during pregnancy.

D4. I adhere to the principles of activity and proper condition during pregnancy.

D5. I take pregnancy supplements as prescribed by the doctor or midwife.

D6. I consult with the doctor or midwife for taking any type of medication during pregnancy (chemical and herbal).

D7. I attend for prenatal care (examinations) as scheduled.

D8. I perform ultrasound and tests in pregnancy recommended by healthcare professionals such as doctor or midwife.

D9. I monitor and control the weight gain during pregnancy.

D10. I use the appropriate methods of sexual relation during pregnancy.

D11. I avoid taking actions that are harmful to pregnancy.

D12. I see the doctor or midwife as soon as possible when any signs of danger in pregnancy are observed.

D13. I ask the doctor or midwife for further explanation if the information and recommendations are not clear enough.

D14. I participate in decision-making about pregnancy issues with the doctor or midwife (providing personal opinions).

D15. I pay attention to the accuracy and appropriateness of information given to other pregnant women.
